# Comparison of the clinical efficacy of unilateral and bilateral pedicle screw short-segment fixation and fusion in the treatment of atlantoaxial fracture-dislocation

**DOI:** 10.3233/THC-220721

**Published:** 2023-09-15

**Authors:** Zhuo Ma, Yan-Nan Zhang, Xun Ma, Chen Chen, Hao-Yu Feng

**Affiliations:** Department of Orthopedic Surgery, Shanxi Bethune Hospital, Shanxi Academy of Medical Sciences, Tongji Shanxi Hospital, The Third Hospital of Shanxi Medical University, Taiyuan, Shanxi, China

**Keywords:** Atlantoaxial, fracture-dislocation, unilateral, pedicle screw, fusion

## Abstract

**BACKGROUND:**

Few studies have compared the clinical efficacy of unilateral and bilateral pedicle screw fixation and fusion in treating atlantoaxial fracture-dislocation.

**OBJECTIVE:**

To compare the efficacy of unilateral and bilateral fixation and fusion for atlantoaxial fracture-dislocation and to explore the feasibility of the unilateral surgical procedure.

**METHODS:**

Twenty-eight consecutive patients with atlantoaxial fracture-dislocation were included in the study from June 2013 to May 2018. They were divided into a unilateral fixation group and a bilateral fixation group (14 patients in each group with an average age of 43.6 ± 16.3 years and 51.8 ± 15.4 years, respectively). The unilateral group had a unilateral anatomical variation of the pedicle or vertebral artery, or traumatic pedicle destruction. All patients underwent atlantoaxial unilateral or bilateral pedicle screw fixation and fusion. Intraoperative blood loss and operation time were recorded. The visual analog scale (VAS) and Japanese Orthopedic Association (JOA) scoring systems were used to evaluate pre- and postoperative occipital-neck pain and neurological function. X-ray and computerized tomography (CT) were used to assess atlantoaxial stability, the implants’ position, and bone graft fusion.

**RESULTS:**

All patients were followed up for 39–71 months postoperatively. Intraoperatively, no spinal cord or vertebral artery injury was observed. At the last follow-up, occipital-neck pain and neurological function in the two groups were significantly improved (P< 0.05). The X-ray films and CT showed satisfactory atlantoaxial stability, implant position, and osseous fusion in all the patients at 6 months postoperatively.

**CONCLUSION:**

Unilateral and bilateral pedicle screw fixation and fusion can restore atlantoaxial stability and improve occipital-neck pain and neurological function in patients with atlantoaxial fracture-dislocation. The unilateral surgical procedure can be a supplementary option for patients with unilateral abnormal atlantoaxial lesions.

## 1. Introduction

Atlantoaxial fracture-dislocation can cause occipital-neck pain, limited neck mobility, and high cervical spinal cord impairment, which seriously affects patients’ quality of life or may result in death [1]. The goal of surgery is decompression, fixation and fusion, and functional reconstruction. Therefore, early and proper surgical selection is critical for the reduction, bone healing, and neurological recovery of patients with atlantoaxial fracture-dislocation [2, 3, 4]. At present, posterior atlantoaxial screw fixation and bone graft fusion are commonly performed in patients with atlantoaxial fracture-dislocation because of their definite clinical effects [5, 6, 7]. However, it is difficult and risky to proceed with this surgery, especially for atlantoaxial anatomical deformities, such as unilateral high straddles, stenosis, occlusion of the vertebral artery, or unilateral dysplasia of the pedicle. It is easy to impair the spinal cord and vertebral artery on the dominant side if the bilateral screws are implanted improperly. The abnormal vertebral artery has poor compensatory ability, leading to insufficient blood supply to the brain and serious consequences [2]. Therefore, unilateral pedicle screw short-segment fixation and fusion is a supplemental procedure for treating atlantoaxial fracture-dislocation with an anatomical variation. However, few studies have compared the clinical efficacy of unilateral and bilateral pedicle screw fixation and fusion in treating atlantoaxial fracture-dislocation. Whether the unilateral procedure can achieve satisfactory results is yet to be explored [8, 9, 10, 11, 12]. This study compares the two surgical methods’ clinical results to examine the feasibility of the unilateral procedure.

## 2. Materials and methods

### 2.1 General data

A retrospective analysis of 28 patients with atlantoaxial fracture-dislocation treated in the orthopedic department from June 2013 to May 2018 was conducted. According to the existence of an atlantoaxial anatomical variation and surgery type, the patients were divided into: 1) a unilateral pedicle fixation group (unilateral group: 14 patients, including 9 males and 5 females, with an average age of 43.6 ± 16.3 years); and 2) a bilateral pedicle fixation group (bilateral group: 14 patients, including 13 males and 1 female, with an average age of 51.8 ± 15.4 years). The causes of injury were traffic injury in 11 cases, sports injury in 8 cases, high-fall injury in 6 cases, and blow injury in 3 cases. Injury type: Anderson type II odontoid fracture in 7 cases, old odontoid fracture in 1 case, odontoid fracture combined with atlantoaxial dislocation in 4 cases, traumatic atlantoaxial dislocation in 11 cases, congenital atlantoaxial dislocation in 1 case, atlantoaxial dislocation with odontoid nonunion in 2 cases, and odontoid combined with anterior arch of atlas fracture in 2 cases. All patients had different degrees of high cervical spinal cord injury symptoms, including occipital-neck pain, limited motion, and numbness and weakness of the limbs, and were treated with posterior atlantoaxial unilateral or bilateral pedicle screw fixation and bone graft fusion. This study was approved by the hospital medical ethics committee.

The inclusion criteria were: patients with typical clinical manifestations and complete imaging data; definite surgical indications; and unilateral anatomical variation of the atlantoaxial (unilateral group).

The exclusion criteria were: history of previous atlantoaxial surgery; atlantoaxial tumor or infectious disease; irreducible atlantoaxial dislocation; severe osteoporosis; bilateral fixation using other surgical methods; combination with motor neuron disease; and poor compliance.

### 2.2 Preoperative preparation

Preoperatively, anteroposterior, mouth-opened, lateral X-ray, computerized tomography (CT), and three-dimensional (3D) reconstruction were performed to evaluate the atlantoaxial fracture and dislocation as well as the shape and diameter of the pedicles. Magnetic resonance imaging (MRI) was performed to evaluate the rupture of the transverse ligament of the atlas, the degree of spinal cord compression, and intramedullary hyperintensity. CT angiography (CTA) was performed to assess the variation and course of the vertebral artery. All patients were examined by a spine surgeon, and the goal was to maintain the cervical spine’s stability. According to the imaging data, a 3D atlantoaxial anatomical model of each patient was printed to determine the surgical strategy. Cranial ring traction reduction was routinely used, and a bedside lateral X-ray of the cervical spine was performed to evaluate the reduction situation. The vital signs of all patients were recorded, and symptomatic treatment, such as nerve dehydration, analgesia, and sputum suction, was provided to patients with spinal cord injuries. Tracheotomy should be performed for those who have difficulty breathing, if necessary.

### 2.3 Surgical procedures

**Figure 1.**
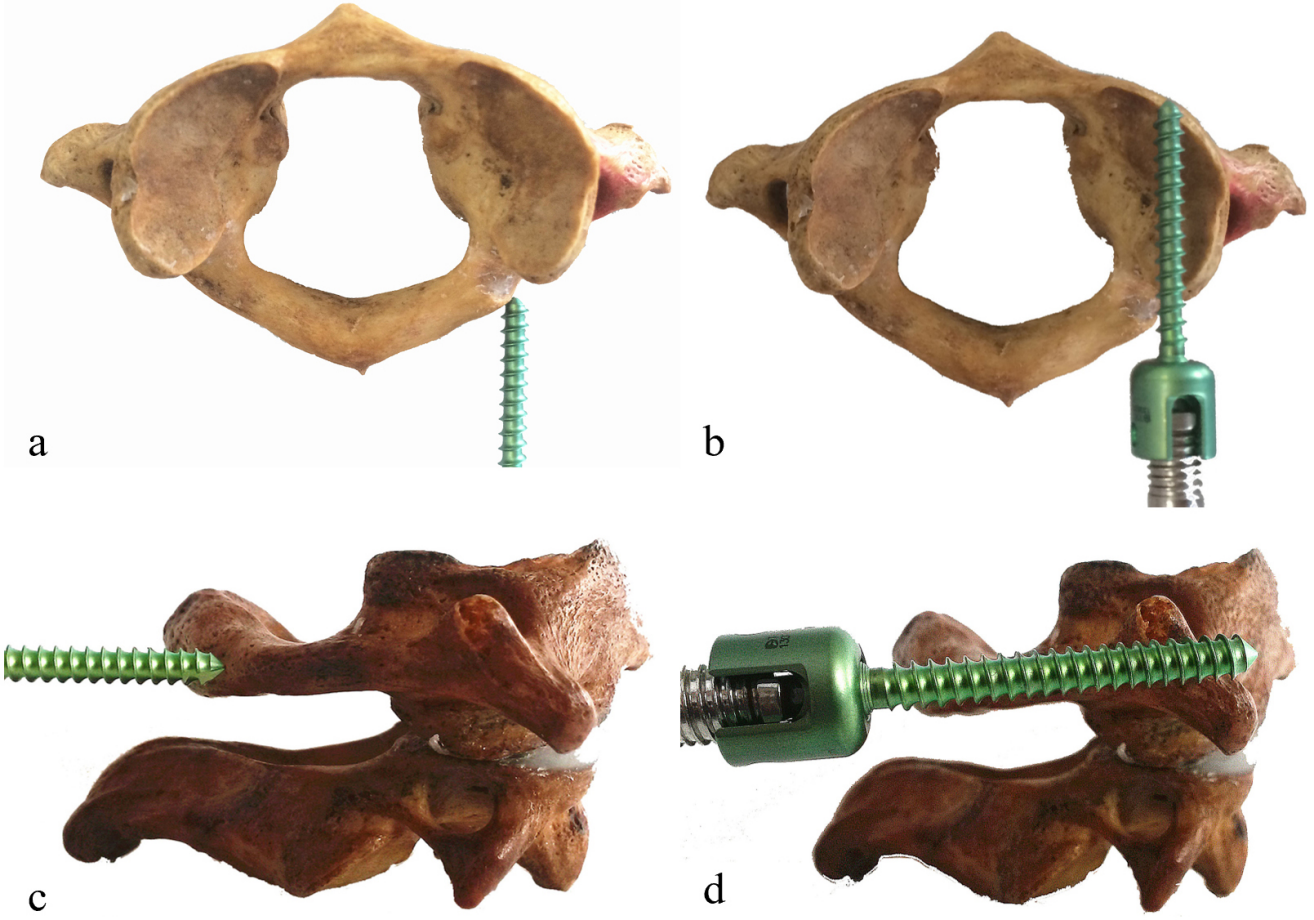
A, B: The entry point and direction of the C1 pedicle screw in horizontal view. C, D: The entry point and direction of the C1 pedicle screw in sagittal view.

**Figure 2.**
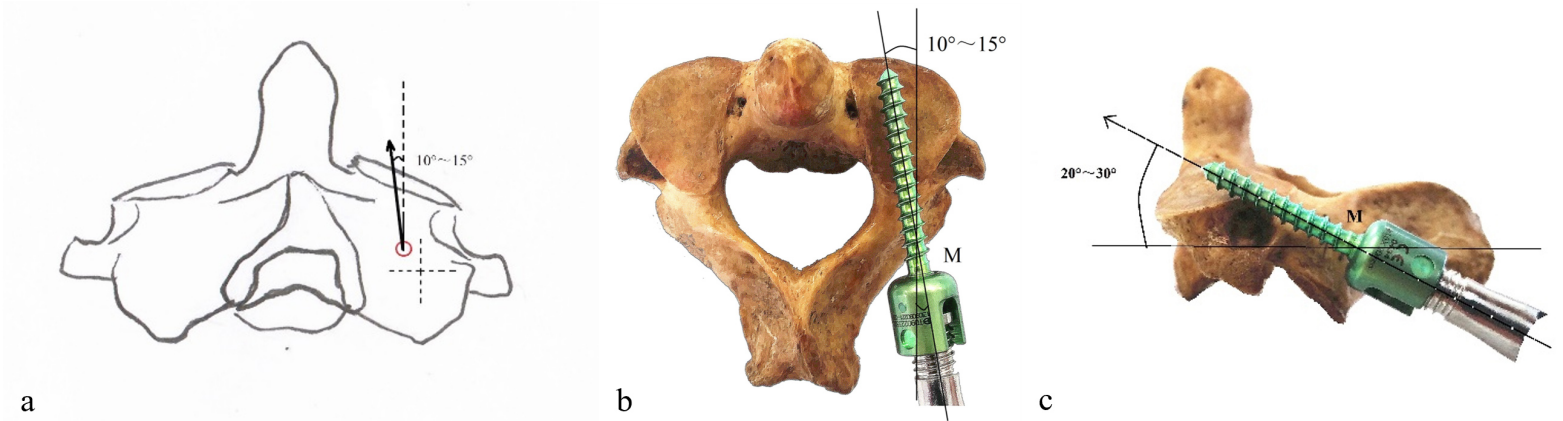
A: The entry point of the C2 pedicle screw. B, C: The insertion angle of the C2 pedicle screw in horizontal and sagittal view.

**Figure 3.**
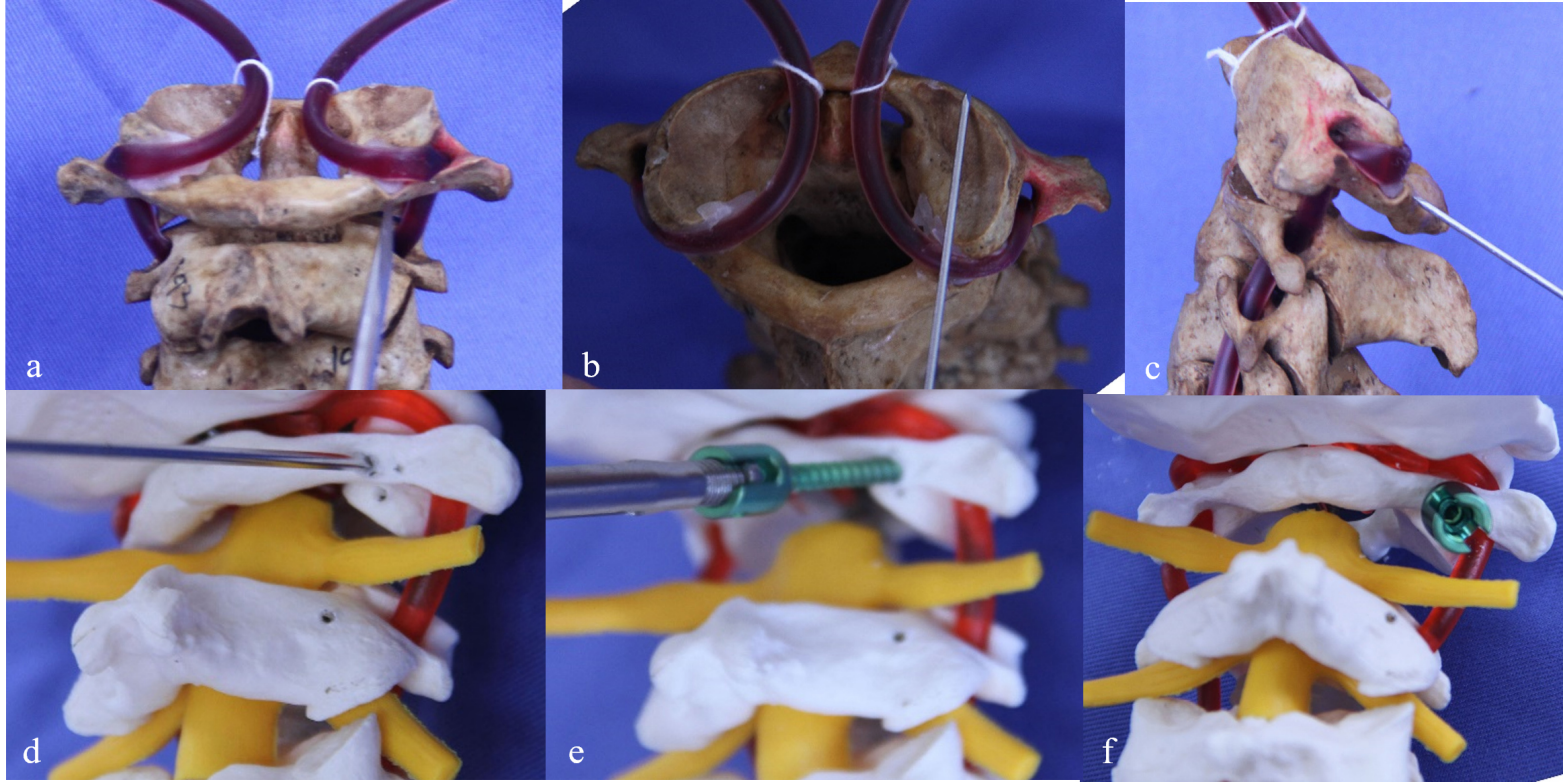
A–C: The entry point and direction of the C1 pedicle screw in the specimen. D–F: The C1 pedicle screw guide pin and pedicle screw were inserted into the 3D printed cervical model.

**Figure 4.**
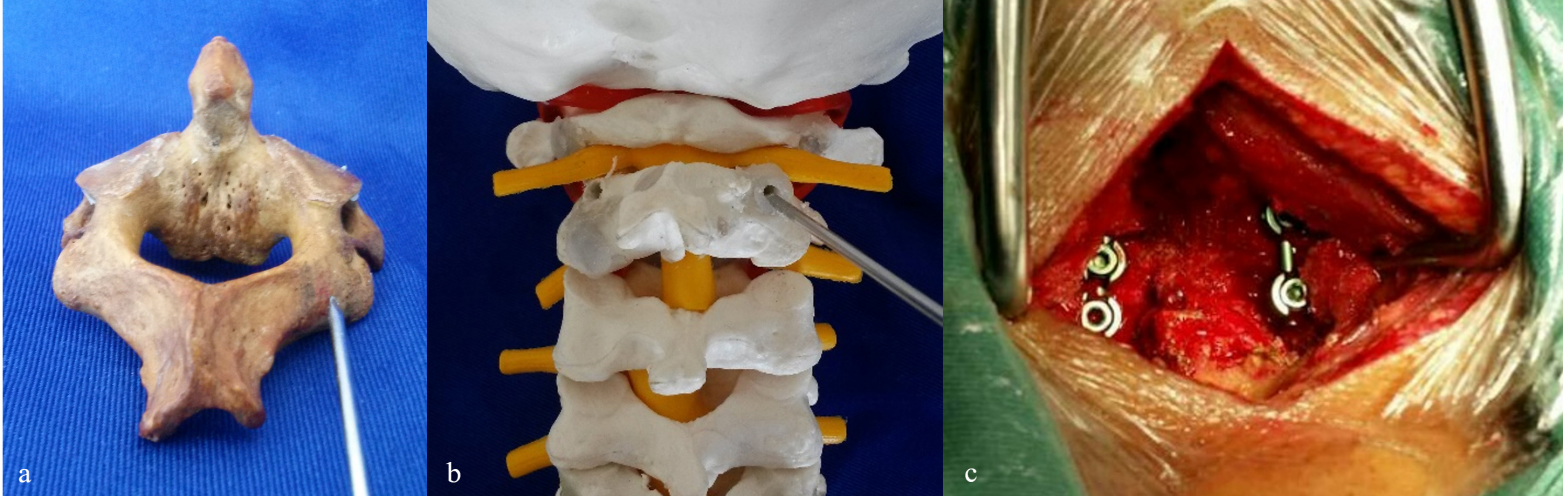
A, B: The entry point and direction of the C2 pedicle screw in the specimen and 3D printed model. C: Intraoperative placement of the C1–2 pedicle screws and connecting rods.

Under general anesthesia, patients were placed in the prone position with appropriate neck flexion and continuous cranial ring traction or fixed by a Mayfield frame. The fluoroscopic evaluation was performed before the incision. An incision was made in the middle of the posterior occipital neck. From the occipital carina to the C3 spinous process, the C1 posterior arch, the C2 spinous process, the lamina, and the pedicle were gradually exposed. The experience of atlantoaxial pedicle screw placement summarized in the previous experiment was as follows [6, 7, 13]: the entry point of the C1 pedicle screw was located at the posterior arch 18–20 mm beyond the midpoint of the C1 posterior tubercle, the intersection of the horizontal line 2 mm above the lower edge of the posterior arch, and the vertical line at the center of the C1 lateral mass with the vertical screw entry in the horizontal plane and inclined upward by 6${}^{\circ }$ in the sagittal plane. After drilling the cortex, the guide needle was placed into the pedicle, the depth was probed, and the 3 mm pedicle screw was inserted after tapping. The entry point of the C2 pedicle screw was located at the center of the upper medial quadrant on the surface of the C2 isthmus. The screw entry angle was 10${}^{\circ }$–15${}^{\circ }$ inward and 20${}^{\circ }$–30${}^{\circ }$ upward (Figs 1 to 4).

Among the 14 patients in the unilateral group, 6 patients had unilateral stenosis or a high-riding course of the vertebral artery, 3 patients had unilateral slender pedicles of dysplasia, 3 patients had intraoperative unilateral hemorrhage of the venous sinus, and hemostasis was performed by gelatin sponge packing, and, in 2 patients, the unilateral C2 pedicles were destroyed due to traumatic atlantoaxial fracture, so the screws could not be placed.

To restore the atlantoaxial and decompress the spinal cord, C2 nerve roots were properly pulled away for protection, and Kocher forceps were used to pull the C1 posterior arch or depress the C2 lamina when there was a C1 anterior dislocation, while the C2 spinous process was pulled backward when there was a C1 posterior dislocation. The rod and top wire were implanted. Afterward, the C1 posterior arch, the C2 spinous process, and the lamina were decortified with a high-speed drill to prepare the bone grafting bed. The autologous posterior superior iliac spinous cancellous bone fragments were mixed into a bone paste and implanted in the bone grafting bed. An indwelling negative pressure drainage tube was placed. All patients underwent intraoperative real-time fluoroscopy to assess the reduction of atlantoaxial dislocation and screw placement.

### 2.4 Postoperative management

Postoperatively, all patients had unobstructed airways, and the drainage tube was removed when the incision drainage volume was less than 40 ml. Lateral and mouth-opened reviewed X-ray was performed. Patients with good general conditions wore neck collars for rehabilitative exercise 2 days after surgery. A neck collar was worn for 2 months to prevent neck flexion, extension, and rotation before osseous fusion in the bone graft area. Outpatient follow-up of the patients was performed at 1, 3, 6, and 12 months postoperatively and every year thereafter; the atlantoaxial reduction, bone graft fusion, and implant positions were evaluated by routine X-ray and CT.

### 2.5 Evaluation index

The intraoperative blood loss and operation time were recorded. The Visual Analog Scale (VAS) and Japanese Orthopedic Association (JOA) scoring systems were applied pre- and postoperatively to evaluate the two groups’ occipital-neck pain and neurological function. The neurological recovery rate was calculated as follows: JOA recovery rate = (postoperative score - preoperative score)/(17 - preoperative score) × 100%.

### 2.6 Statistical analysis

SPSS 22.0 statistical software was used to test the normality of the quantitative indices. Statistical descriptions were performed as the mean ± standard deviation, and a t-test with paired samples was used. For measurement data that did not conform to a normal distribution, the rank sum test was used. The test level P< 0.05 was considered statistically significant.

**Figure 5.**
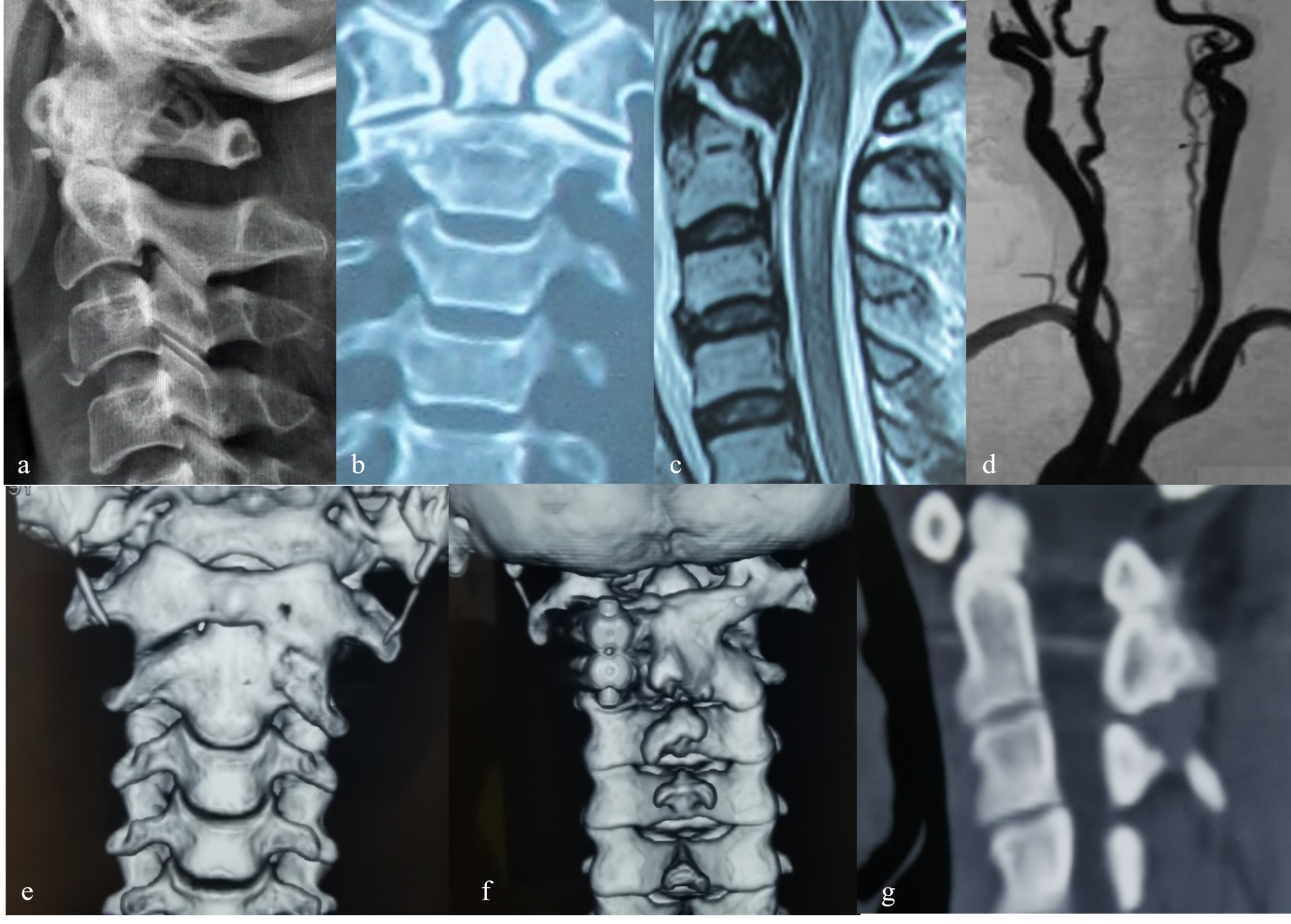
Female, 47 years old, odontoid fracture caused by traffic injury leading to cervical spinal cord injury and quadriplegia in the unilateral group. A, B: Preoperative lateral X-ray film and coronal CT showed Anderson type II odontoid fracture. C: Preoperative MRI T2WI showed odontoid fracture and diffuse intramedullary hyperintensity at C2 level. D: Preoperative CTA showed obvious stenosis of the vertebral artery on the right side. E: The anteroposterior X-ray film on the 2${}^{\text{nd}}$ day after surgery showed the C1–2 pedicle screws and rods on the right side were in good position. F: The sagittal CT at 6 months after surgery showed the bone graft between the C1 posterior arch and C2 spinous process was clear and fused. G: The axial CT at 6 months after surgery showed the pedicle screw on the right side was in good position without loosening or rupture.

## 3. Results

**Figure 6.**
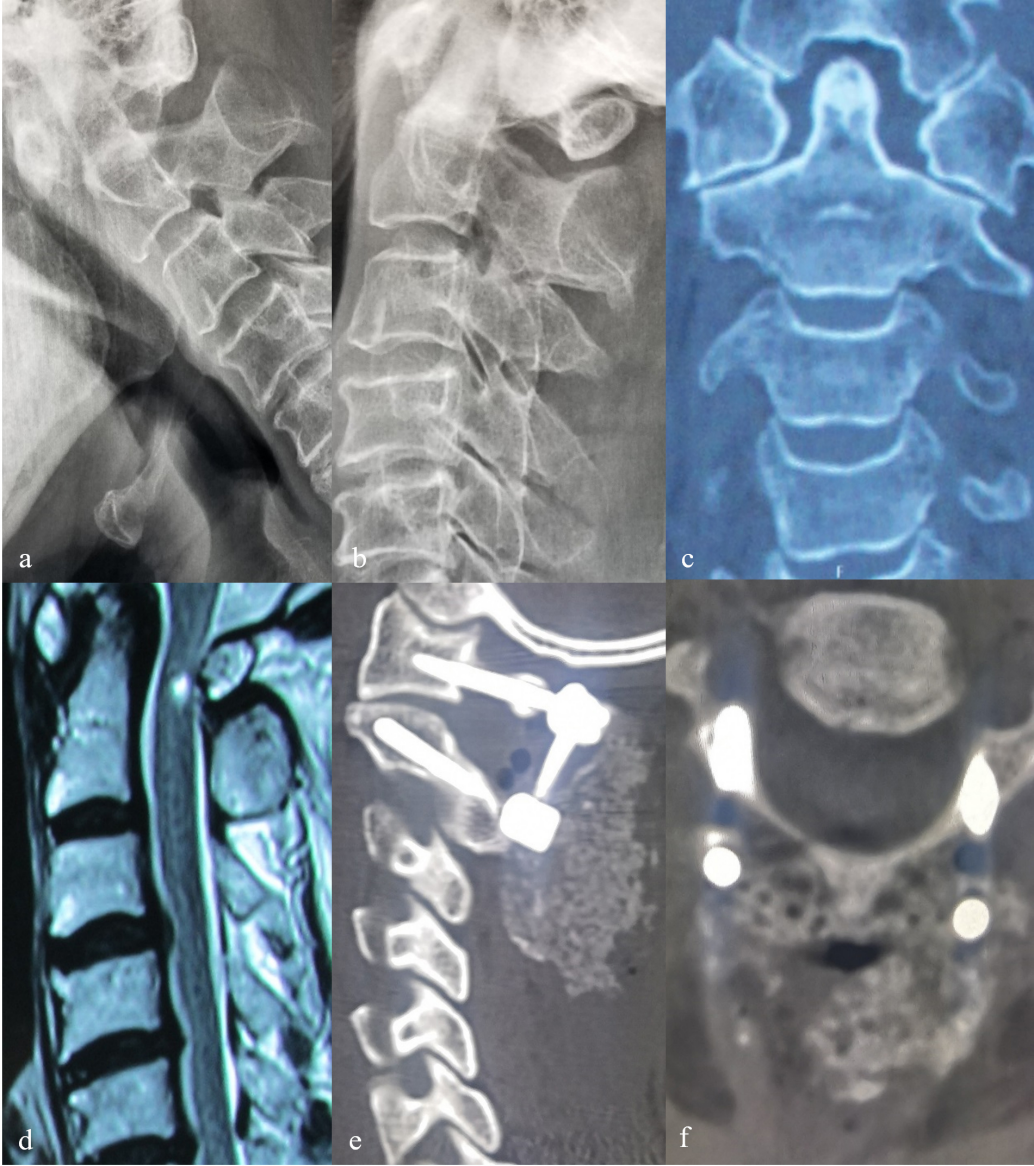
Male, 68 years old, atlantoaxial dislocation caused by high-fall injury leading to a cervical spinal cord injury and quadriplegia in the bilateral group. A–C: Preoperative dynamic X-ray film and coronal CT showed atlantoaxial dislocation. D: Preoperative MRI T2WI showed intramedullary hyperintensity at the odontoid level. E, F: The sagittal and axial CT at 6 months after surgery showed the implants were in a good position, the bone mass in the bone graft area was sufficient, and the osseous fusion was observed.

The unilateral and bilateral groups were followed up for 50.9 ± 10.8 months and 63.4 ± 22.8 months, respectively (P> 0.05). A total of 42 pedicle screws were successfully implanted in all patients at one time without spinal cord and vertebral artery injury. The venous plexus was injured in 3 patients when the lower edge of the C1 posterior arch was peeled off, and hemostasis was successfully performed by local compression with a medical gelatin sponge. There was no significant difference in the operation time (141.8 ± 28.7 min, 139.3 ± 43.4 min) or intraoperative blood loss (203.6 ± 84.3 ml, 187.9 ± 138.6 ml) between the two groups (P> 0.05). At the final follow-up, occipital-neck pain (VAS score: 0.9 ± 1.9 points, 0.9 ± 1.5 points) and neurological function (JOA score: 15.6 ± 2.2 points, 16.4 ± 1.0 points) in the two groups improved significantly compared to those recorded preoperatively (VAS score: 7.6 ± 0.5 points, 7.3 ± 0.5 points; JOA score: 11.8 ± 3.1 points, 12.2 ± 4.0 points) (P< 0.05). However, no significant difference was observed in the final follow-up JOA recovery rate (59.1 ± 42.0%, 55.4 ± 45.4%) between the two groups (P> 0.05). Only 1 patient had screw loosening after removing the neck collar at 1 month postoperatively, and no implant loosening or breakage was found after timely secondary fixation and fusion. Axial symptoms occurred in 1 patient in each group postoperatively and were relieved gradually within 3–6 months by symptomatic treatment, including pain relief, muscle relaxation, and rehabilitation therapy. The X-ray films and CT showed satisfactory atlantoaxial stability, implant position, and osseous fusion in all patients at 6 months postoperatively (Table 1, Figs 5 and 6).

**Table 1:** Comparison of the general data and clinical outcomes of the two groups

	Unilateral (n= 14)		Bilateral (n= 14)		T	P
Age (years)	43.57	± 16.27	51.79	± 15.42	-1.371	0.182
Operation times (min)	141.79	± 28.663	139.29	± 43.406	-0.180	0.859
Intraoperative blood loss (ml)	203.57	± 84.271	187.86	± 138.629	0.362	0.720
Preoperative VAS scores (neck pain)	7.57	± 0.514	7.29	± 0.469	1.201	0.241
Postoperative VAS scores (neck pain)	0.93	± 1.859	0.86	± 1.512	0.112	0.912
Preoperative JOA scores	11.79	± 3.068	12.21	± 3.945	-0.217	0.830
Postoperative JOA scores	15.64	± 2.170	16.43	± 1.016	-1.227	0.231
Mean follow-up (months)	50.86	± 10.76	63.36	± 22.762	-1.273	0.215
JOA recovery rate (%)	59.05	± 41.98	55.42	± 45.37	-0.023	0.982

## 4. Discussion

### 4.1 Surgical characteristics

At present, posterior bilateral short-segment screw fixation and fusion is the commonly performed surgical technique for atlantoaxial fracture-dislocation because the procedure can achieve strong biomechanical stability and definite clinical effect [5, 6, 7, 8, 9, 10, 11, 12]. However, if bilateral fixation is performed in patients with high straddles, stenosis, and occlusion of the unilateral vertebral artery caused by an anatomical variation, it is easy to impair the vertebral artery on the dominant side, which results in severely insufficient blood supply to the bilateral vertebral artery and leads to serious consequences, such as ischemic hypoxic encephalopathy [2, 14]. Similarly, for patients with a unilateral slender or absent atlantoaxial pedicle due to anatomical dysplasia, the risk of spinal cord and vertebral artery impairment is extremely high if the screws are improperly placed intraoperatively.

In this study, the authors performed posterior unilateral and bilateral pedicle screw short-segment fixation and fusion in 28 patients with atlantoaxial fracture-dislocation. The postoperative long-term follow-up observation showed satisfactory reconstruction of atlantoaxial stability, osseous fusion, and improvements in neurological function and occipital-neck pain.

A study by Paik et al. [15] reported that from the perspective of biomechanics, unilateral C1 lateral mass screw combined with C2 pedicle screw fixation was also an effective technique for patients with atlantoaxial instability and an anatomical variation for whom the screws could not be placed bilaterally. Studies by Hue and Wang et al. [14, 16] reported that for patients with atlantoaxial injury combined with unilateral anatomical deformity of the vertebral artery and pedicle or atlantoaxial comminuted fracture, screw fixation on the dominant side could also achieve favorable atlantoaxial reduction, osseous fusion, and recovery of neck pain and neurological function compared with traditional bilateral atlantoaxial fixation and fusion.

In the authors’ study, the occipital-neck pain and neurological function of the two groups were significantly improved at the last follow-up compared with those preoperatively. The study results show that unilateral short-segment pedicle screw fixation and fusion was feasible for treating atlantoaxial fracture-dislocation with anatomical variation and achieved similar clinical efficacy as bilateral screw placement, which was similar to the results of the above studies.

### 4.2 Surgical indications and contraindications

Pedicle screw fixation and fusion for atlantoaxial fracture-dislocation is difficult and may injure the spinal cord, vertebral artery, and venous plexus. The surgical indications must be strictly controlled. According to the biomechanical stability and strength of the screws, the C1-2 pedicle screw is recommended as the preferred alternative for atlantoaxial injury [17, 18, 19, 20, 21, 22]. The surgical indications included: 1) atlantoaxial fracture-dislocation; 2) odontoid fracture or nonunion; and 3) traumatic or rheumatoid atlantoaxial instability [17, 18, 23]. The contraindications included 1) irreducible atlantoaxial dislocation; 2) traumatic atlantoaxial instability with C2 pedicle fracture; 3) severe osteoporosis; and 4) atlantoaxial instability due to tumor or infectious disease [17, 18, 23]. Meanwhile, unilateral pedicle screw fixation was used as a “salvage” option [24], and the specific indications included: 1) dysplasia, stenosis, and absence of unilateral C1 posterior arch and C2 pedicle; 2) stenosis, occlusion, and abnormal course of unilateral vertebral artery; 3) comminuted fracture of unilateral C1 lateral mass, posterior arch, and C2 pedicle; and 4) severe unilateral venous sinus hemorrhage resulting in difficulty of bilateral screw placement. In this study, the authors’ reasons for the unilateral surgical procedure were based on the specific indications above.

### 4.3 Main surgical points

It is essential to ensure the safety and accuracy of screw placement, reduce surgical complications, and improve the osseous fusion rate because of the special anatomical structure of the atlantoaxial vertebra, the complexity of the adjacent relationship between peripheral blood vessels and nerves, and the high incidence of anatomical variation. To achieve satisfactory clinical efficacy, there must be complete perioperative management, including the following: 1) A detailed preoperative plan. Strict imaging evaluation (anteroposterior, mouth-opened, lateral X-ray, CT, 3D reconstruction, MRI, CTA, and 3D printing model) was performed to identify the size and developmental state of the C1 lateral mass, posterior arch and C2 pedicle, the course and variation of the vertebral artery, the severity and extent of the fracture-dislocation, and the degree of spinal cord compression. Based on the evaluation above combined with intraoperative fluoroscopy, the precise screw entry point, screw entry angle, and screw length for individualized screw placement were determined. If atlantoaxial reduction cannot be performed preoperatively, a further reduction can be performed during the operation by temporary cranial ring traction or using the Mayfield head frame under general anesthesia. 2) Intraoperatively, the C1 posterior arch and C2 pedicle must be exposed carefully, and the venous plexus must be dissected gently and bluntly. When venous plexus hemorrhage occurs, local compression and packing with a medical gelatin sponge can be used for hemostasis. 3) When the pedicle screw is placed, the junction of the C1 posterior arch, the midpoint of the lateral mass, and the upper inner quadrant of the C2 isthmus should be identified. After accurately determining the screw entry point, the drill hole was opened under direct vision. 4) The cortical bone should be polished with a burr. The bone graft bed (C1 posterior arch and C2 spinous process-laminae) should be treated delicately. The cancellous bone obtained from the autologous iliac bone was mixed into a bone paste for sufficient bone grafting. 5) A neck collar should be worn for 3 months, or longer if necessary, to restrict neck movement before bony fusion is achieved in the bone grafting area. Otherwise, there is a risk of the implant loosening, loss of reduction, and nonfusion of the graft.

The authors’ experience is as follows: for patients with a unilateral abnormal vertebral artery, the abnormal side is selected for screw placement, which can preserve the blood supply of the vertebral artery on the normal side and prevent the blood supply on the abnormal side from being unable to compensate due to the damage of the vertebral artery on the normal side. This can lead to serious vertebral artery injury and even serious complications, such as ischemic hypoxic encephalopathy and cerebral infarction. For patients with unilateral dysplasia of the atlantoaxial pedicle, the normal side is selected for screw placement, which can effectively reduce the difficulty of screw placement and reduce the risk of spinal cord and vertebral artery injury.

## 5. Conclusion

Medium- and long-term follow-up showed that unilateral and bilateral pedicle screw fixation and fusion restored atlantoaxial stability and improved occipital-neck pain and neurological function in patients with atlantoaxial fracture-dislocation. The unilateral surgical procedure can be a supplementary option for patients with unilateral abnormal atlantoaxial lesions.

## Funding

Science Foundation of Shanxi Health Commission (No. 2018009) funds were received in support of this work.

## Ethics approval

The study was approved by the Ethics Committee of Shanxi Bethune Hospital.
